# Association between urinary glyphosate levels and hand grip strength in a representative sample of US adults: NHANES 2013–2014

**DOI:** 10.3389/fpubh.2024.1352570

**Published:** 2024-02-21

**Authors:** Yu-Wei Fang, ChiKang Wang, Chien-Yu Lin

**Affiliations:** ^1^Division of Nephrology, Department of Internal Medicine, Shin-Kong Wu Ho-Su Memorial Hospital, Taipei, Taiwan; ^2^School of Medicine, College of Medicine, Fu Jen Catholic University, New Taipei, Taiwan; ^3^Department of Environmental Engineering and Health, Yuanpei University of Medical Technology, Hsinchu, Taiwan; ^4^Department of Internal Medicine, En Chu Kong Hospital, New Taipei City, Taiwan

**Keywords:** glyphosate exposure, herbicide effects, muscle strength, National Health and Nutrition Examination Survey (NHANES) data analysis, muscle mass assessment, sarcopenia risk factors

## Abstract

**Introduction:**

Glyphosate, a widely utilized herbicide globally, has been linked to various health issues, including cancer, birth abnormalities, and reproductive issues. Additionally, there is growing experimental support indicating potential harm to skeletal muscles. Despite this, the impact of glyphosate on human muscle health remains unclear.

**Methods:**

We examined information gathered from the 2013-2014 National Health and Nutrition Examination Survey (NHANES), which included 1466 adults aged 18 or older. Our primary aim was to investigate the relationship between glyphosate exposure and hand grip strength, as well as its influence on lean muscle mass.

**Results and discussion:**

Our investigation uncovered a detrimental correlation between glyphosate exposure and all measures of grip strength, except for the second test of the first hand. Specifically, we observed a statistically significant adverse association between glyphosate exposure and combined grip strength, which is calculated as the sum of the highest readings from both hands (ß coefficient of −2.000, S.E. = 0.891, *p* = 0.040). We did not observe a significant correlation between glyphosate levels, lean muscle mass, and the likelihood of reaching maximum grip strength meeting sarcopenia criteria. Additionally, we observed an interaction between age and glyphosate, as well as between body mass index (BMI) and glyphosate, concerning the association with combined grip strength. In this comprehensive analysis of NHANES data, our study reveals a potential association between glyphosate exposure and hand grip strength in the adult population. Our findings suggest the need for deeper exploration into the health effects of glyphosate exposure and its impact on muscle strength, shedding light on possible public health concerns.

## 1 Introduction

Glyphosate, which has been the active component in herbicides since 1974, works as a chemical that disrupts the shikimate pathway, a metabolic pathway used by plants to synthesize essential aromatic amino acid (1). Glyphosate-based herbicides (GBH) are a combination of glyphosate and surfactants that amplify its permeation into plants and augment its efficacy (2). Glyphosate and GBH are widely used due to their exceptional efficacy in managing weed proliferation, rendering them the most extensively utilized herbicides worldwide (3). Individuals may potentially come into contact with these two chemicals through various routes, including skin, inhalation, and oral consumption (4). Although glyphosate was once thought to be safe in animals, increasing apprehension has arisen in recent years regarding possible negative health effects associated with glyphosate and GBH. Numerous investigations have established connections between glyphosate exposure and a range of health concerns, such as cancer, birth abnormalities, endocrine, and reproductive issues (5, 6). As a result of these findings, the International Agency for Research on Cancer classified glyphosate as a probable human carcinogen (7).

Glyphosate and GBH have been found to have demonstrated varying impacts on different types of cells, depending on the concentration levels used during testing (1). Moreover, research has shown that these herbicides can cause cytotoxicity and genotoxicity in human cell cultures in a dose-dependent manner, even at environmentally relevant concentrations (8). Experimental research has examined the influence of glyphosate and GBH on skeletal muscle. Glyphosate exposure was found to decrease energy reserves (9, 10), alter acetylcholinesterase enzyme activity (11), change muscle morphology and functioning (12), and reduce muscle strength (13). However, results from these studies were inconsistent (14, 15).

While experimental research has yielded evidence suggesting that glyphosate and GBH could have adverse impacts on skeletal muscle, their connection in humans remains inadequately explored. It’s worth highlighting that there is a lack of studies examining the potential link between glyphosate exposure and the well-being of skeletal muscles in the general human population representing a country. Dynamometry is a reliable, valid, and responsive method for measuring muscle strength (16, 17). To assess lean muscle mass, dual-energy X-ray absorptiometry (DXA) is widely recognized as the gold standard for measuring body composition. Lean body mass excluding bone mineral content is a useful measure for evaluating lean muscle mass (18). To address this knowledge gap, we analyzed data from the National Health and Nutrition Examination Survey (NHANES) conducted between 2013 and 2014. The dataset offers information on urinary glyphosate levels, hand grip strength tests, and lean muscle mass measured by DXA. Our study solely examined the adult population since hand grip strength and lean body mass can significantly differ among adults and children. Furthermore, adults may have a higher incidence of underlying medical conditions such as diabetes and chronic kidney disease that must be accounted for in the analysis (19, 20). Limiting our study to adults allowed us to better control for these variables and gain a clearer understanding of the effects of glyphosate. Our study aimed to enhance our comprehension of the association between glyphosate levels and muscle health in the general adult population by examining the relationship between urinary glyphosate levels, hand grip strength, and lean muscle mass.

## 2 Materials and methods

### 2.1 Study population

**Figure 1:**
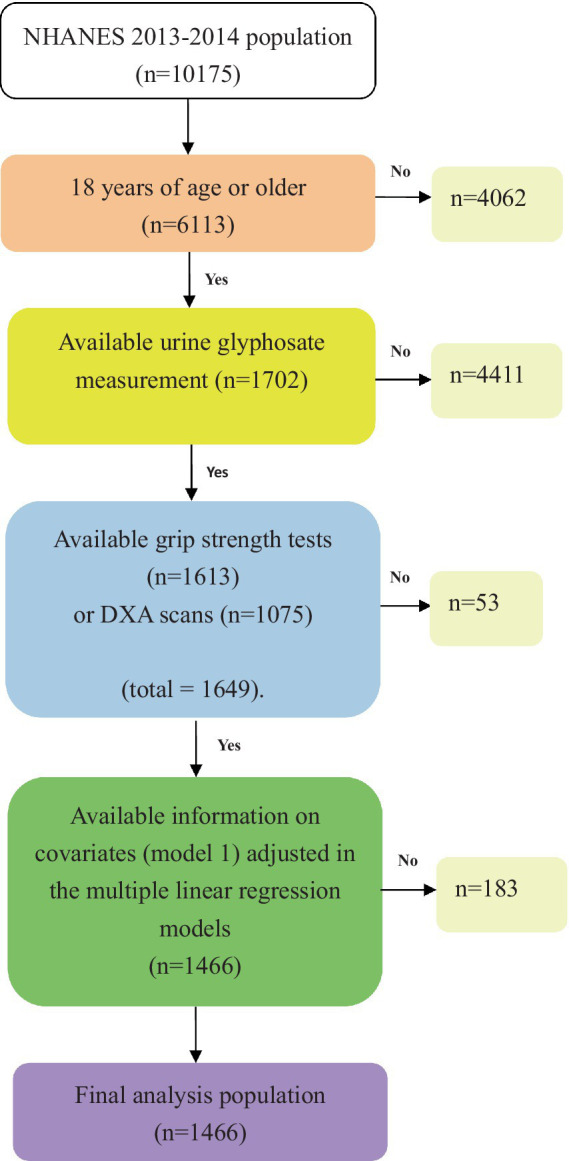
Flow chart algorithm.

The NHANES is a biennial nationwide survey that recruits a representative sample of the general population in the United States. Detailed information on the survey methodology and consent forms can be found on the NHANES website (21). In the current study, we utilized the NHANES 2013–2014 database and constrained population to individuals who were 18 years or older, possessed available measurements of glyphosate exposure, and pertinent demographic data. In addition, we excluded those who lacked measurements of hand grip strength or lean muscle mass. Our final study sample comprised 1,466 subjects, and a flow chart detailing the algorithm can be observed in Figure 1.

### 2.2 Measurement of urinary glyphosate levels

In the NHANES 2013–2014 study, urinary glyphosate levels were evaluated in a subgroup consisting of one-third of participants aged 6 years and above. Our analysis focused on data collected from individuals who were 18 years of age or older. The techniques used for measuring glyphosate levels have been documented in prior publications (22). For glyphosate levels that fell below the limits of detection (LOD), NHANES provided an imputed value, which was calculated as LOD divided by the square root of 2. The analytical methodology employed in the study is available on the NHANES website (23).

### 2.3 Measurement of muscle strength – hand grip strength test

NHANES 2013–2014 evaluated the strength of participants’ hand grips, aged 6 and above while excluding those who had undergone hand or wrist surgery within the past 3 months or were unable to grip the dynamometer with both hands, through the use of a dynamometer. We collected data from individuals aged 18 and over in this study. Each participant proceeded to squeeze the dynamometer as forcefully as feasible with one hand, followed by the other hand. Three repetitions were conducted for each hand, with a 60-s rest interval between measurements of the same hand. Combined grip strength was calculated as the sum of the highest readings obtained from three attempts on each hand. Hand strength is an essential diagnostic measure to identify sarcopenia, a condition characterized by weak muscles. Grip strength cutoff points of less than 30 kg in men and less than 20 kg in women are indicative of low muscle strength (24). For further instructions, please refer to the NHANES website (25).

### 2.4 DXA – lean body mass

The NHANES DXA scan offers a comprehensive assessment of body composition and is performed on the entire body. Individuals between the ages of 8 and 59 years were eligible, except for those who were pregnant, had recently received radiographic contrast material, or exceeded the weight or height limit of the DXA table. This study included data from individuals aged 18 and over to assess the relationship between glyphosate exposure and muscle mass in adults. We used lean body mass, which excludes bone mineral content, as a measure to evaluate lean muscle mass. The examination protocol details can be found in the NHANES website (26).

### 2.5 Covariates

According to the NHANES website, proficient personnel at all study sites employed uniform procedures to collect data. During the household interview, data on sociodemographic factors like age, gender, and race/ethnicity were collected. After analyzing the responses to the smoking questionnaire, participants were sorted into one of three categories: active smokers, exposed to environmental tobacco smoke (ETS), or non-smokers (27). The alcohol consumption questionnaire determined whether a participant had consumed at least 12 alcoholic beverages in the past year, and the responses were then divided into two categories. Total energy and total protein intake calculations involved averaging data from 2 days of dietary intake questionnaires. Physical activity was assessed by adding up the scores of different activities and multiplying them by their corresponding metabolic equivalent of task scores, as recommended by the NHANES website (28). For this study, chronic kidney disease was characterized as an estimated glomerular filtration rate below 60 mL/min per 1.73 square meters (29). Diabetes mellitus was defined as a fasting serum glucose level ≥126 mg/dL, or a glycated hemoglobin ≥6.5% or the self-reported current use of anti-hyperglycemic medications. Additionally, potential confounders such as body mass index (BMI), urinary creatinine, and diabetes mellitus were considered in this study.

### 2.6 Statistics

The investigation displayed glyphosate concentrations in units of μg/L or μg/g creatinine. Comparisons of geometric means between groups were carried out through the two-tailed Student’s t-test and one-way analysis of variance. The sampling weights used were in accordance with protocols specified on the NHANES website (30). To evaluate the association between urinary glyphosate levels, hand grip strength, and lean body mass, a complex sample of the general linear model was employed. To investigate whether there is clinical significance in understanding the relationship between glyphosate and muscle strength, we employed a complex sample of the logistic regression analysis to explore a potential link between glyphosate concentrations and the presence of sarcopenia. Sarcopenia was defined as having a maximal grip strength assessment of less than 30 kg in males and less than 20 kg in females. For the purpose of covariate adjustment, two distinct models were utilized. In Model 1, age, gender, ethnicity, BMI, smoking, alcohol consumption, household income, and urinary creatinine were adjusted. Model 2, in addition to the adjustments made in Model 1, also took into account total energy intake, total protein intake, physical activity, chronic renal failure, and diabetes mellitus. Instead of being adjusted for hydration, urinary creatinine was treated as an independent variable based on previous research (31). The analysis utilized the natural logarithm (ln) of glyphosate and urinary creatinine owing to their non-Gaussian distributions. The statistical examination was carried out using SPSS version 20 (SPSS Inc., Chicago, Illinois, United States), and the significance level was set at *p* < 0.05 to determine statistical significance.

## 3 Results

The study sample had a mean age of 48.16 years (SD = 18.31). Among the participants, 80.2% had detectable concentrations of glyphosate, with a mean of 0.55 μg/L (SD = 0.54). Table 1 presents the geometric means of glyphosate for different subgroups, indicating elevated urinary glyphosate levels among men, older individuals, non-Hispanic Black participants, and those with a higher BMI. Additionally, after adjusting for creatinine, glyphosate levels were higher in women, older individuals, non-Hispanic white participants, and non-smokers.

**Table 1:** The geometric means (S.E.) of urinary glyphosate levels in different demographic subgroups.

	No.	Glyphosate (μg/L)	*p* value	Glyphosate (μg/g creatinine)	*p* value
Total	1,466	0.40 (1.02)		0.43 (1.02)	
Gender			0.003		<0.001
Men	710	0.43 (1.03)		0.37 (1.03)	
Women	756	0.38 (1.03)		0.49 (1.03)	
Age (years)			<0.001		<0.001
18–40	521	0.38 (1.03)		0.34 (1.03)	
40–59	486	0.37 (1.03)		0.43 (1.03)	
≥ 60	459	0.47 (1.04)		0.55 (1.04)	
Ethnicity			0.001		<0.001
Mexican-American	196	0.38 (1.05)		0.39 (1.06)	
Other Hispanic	127	0.39 (1.07)		0.42 (1.07)	
Non-Hispanic white	692	0.41 (1.03)		0.47 (1.03)	
Non-Hispanic black	257	0.47 (1.05)		0.37 (1.05)	
Non-Hispanic Asian	148	0.34 (1.07)		0.44 (1.07)	
Other ethnicity	46	0.36 (1.10)		0.34 (1.12)	
Household income (USD)			0.342		0.556
< 4,500	679	0.41 (1.03)		0.43 (1.03)	
≥ 4,500	787	0.40 (1.03)		0.43 (1.03)	
Body mass index (kg/m^2^)			0.008		0.440
<25	448	0.37 (1.03)		0.44 (1.04)	
25–30	465	0.41 (1.04)		0.43 (1.04)	
≥30	553	0.43 (1.03)		0.42 (1.03)	
Smoking status			0.083		0.002
Non-smoker	895	0.41 (1.03)		0.45 (1.03)	
ETS	216	0.37 (1.05)		0.39 (1.05)	
Current smoker	355	0.42 (1.04)		0.40 (1.04)	
Alcohol consumption (drink/year)			0.917		0.230
<12	438	0.40 (1.04)		0.44 (1.04)	
≥12	1,028	0.40 (1.02)		0.42 (1.02)	

Tested by Student’s 2-tailed *t*-test or by one-way analysis of variance. ETS, environmental tobacco smoke.

**Figure 2:**
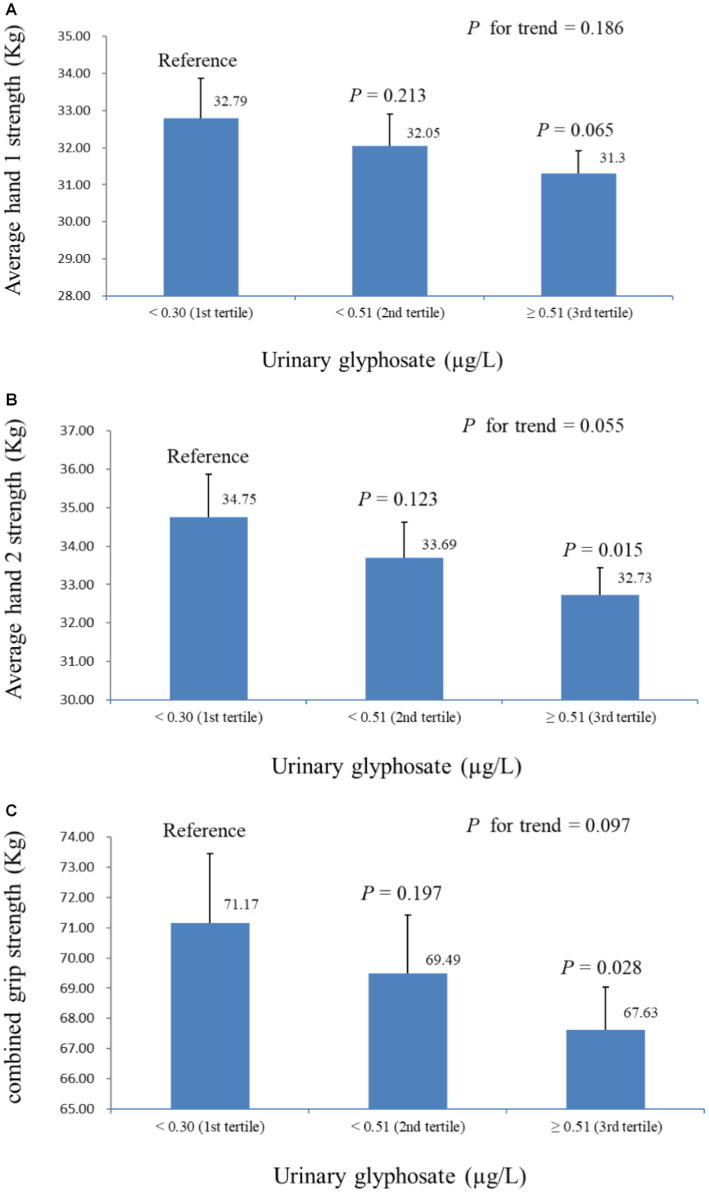
Hand grip strength across tertiles of urine glyphosate in multiple linear regression models (adjusted for model 2), with results weighted for sample strategy. **(A)** Average hand 1 strength. **(B)** Average hand 2 strength. **(C)** Combined grip strength.

Table 2 presents the linear regression coefficients of grip strength with a one-unit increase in ln-urinary glyphosate. Except for test 2 of hand 1, all other grip strength measurements were negatively correlated with ln-glyphosate levels, with ß coefficients of −2.000 (S.E. = 0.891, *p* = 0.040) for combined grip strength. Figure 2 shows an overview of grip strength across urinary glyphosate tertiles in multiple linear regression models. The results indicate that average hand 1, average hand 2, and combined grip strength do not significantly decrease with increasing glyphosate tertiles. However, both average hand 2 and combined grip strength at the highest glyphosate tertile showed a significant reduction compared to the lowest tertile (*p* = 0.015 for average hand 2 strength and *p* = 0.028 for combined grip strength, respectively).

**Table 2:** Linear regression coefficients (S.E.) of grip strength with a unit increase in ln-urinary glyphosate in multiple linear regression models, with results weighted for sampling strategy.

	Ln-glyphosate (μg/L)					
Grip strength (Kg)	Unweighted sample size/population size	Model 1 adjusted *β* (SE)	*p* value	Unweighted no. /Population size	Model 2 adjusted *β* (SE)	*p* value
Hand 1, test 1	1465/210528977	−1.274 (0.458)	0.016	1236/180576129	−1.077 (0.487)	0.043
Hand 1, test 2	1463/210068885	−1.008 (0.445)	0.039	1234/180116037	−0.801 (0.487)	0.121
Hand 1, test 3	1463/210068885	−1.077 (0.386)	0.014	1234/180116037	−0.961 (0.389)	0.026
Hand 1, average	1463/210068885	−1.136 (0.424)	0.017	1234/180116037	−0.965 (0.446)	0.047
Hand 2, test 1	1438/207412005	−1.279 (0.447)	0.012	1212/177702499	−1.081 (0.457)	0.032
Hand 2, test 2	1437/207292371	−1.187 (0.405)	0.010	1211/177582866	−0.919 (0.405)	0.038
Hand 2, test 3	1437/207292371	−1.286 (0.480)	0.017	1211/177582866	−1.137 (0.502)	0.039
Hand 2, average	1437/207292371	−1.248 (0.435)	0.012	1211/177582866	−1.045 (0.444)	0.033
Combined grip^*^	1438/207412005	−2.372 (0.857)	0.014	1212/177702499	−2.000 (0.891)	0.040

Model 1 adjusted for age, gender, ethnicity, smoking status, drinking status, household income, BMI, urinary creatinine. Model 2 adjusted for model 1 plus total energy intake, total protein intake, physical activity, diabetes mellitus, chronic kidney disease. ^*^Combined grip strength: sum of the largest reading from each hand. Ln, natural logarithm.

Table 3 displays the linear regression coefficients of lean muscle mass with a one-unit increase in ln-urinary glyphosate. There is no significant association between glyphosate levels and lean muscle mass (*p* = 0.063 for right arm and *p* = 0.143 for left arm, respectively). In Table 4, the odds ratios of maximal grip strength fulfilling the criteria of sarcopenia with a one-unit increase in ln-glyphosate in logistic regression models are presented. However, glyphosate levels are not statistically significant in relation to the risk of sarcopenia, as defined by maximal hand grip strength. Table 5 depicts the inverse association between glyphosate and the combined grip strength across various subgroups of the research participants. This association was significant in men, individuals aged ≥60, non-Hispanic white, those with lower income, and those with a BMI between 25 and 30. Furthermore, we observed an interaction between age and glyphosate (*p* for interaction = 0.001), as well as between BMI and glyphosate (*p* for interaction = 0.004), concerning the association with combined grip strength.

**Table 3:** Linear regression coefficients (S.E.) of lean muscle mass with a unit increase in ln-urinary glyphosate in multiple linear regression models, with results weighted for sampling strategy.

	Ln-glyphosate (μg/L)					
Lean muscle mass (gm)	Unweighted sample size/population size	Model 1 adjusted *β* (SE)	*p* value	Unweighted no. /population size	Model 2 adjusted *β* (SE)	*p* value
Right arm	965/147581840	−52.783 (27.743)	0.076	806/123894996	−54.976 (27.404)	0.063
Left arm	962/147610237	−55.272 (28.124)	0.068	802/124021746	−44.452 (28.792)	0.143
Right leg	949/145077705	27.935 (71.723)	0.702	793/122340878	42.058 (60.636)	0.499
Left leg	937/142389163	5.261 (74.310)	0.944	784/120211331	27.556 (73.742)	0.714
Trunk	940/143947252	1.927 (167.426)	0.991	783/120888689	−26.506 (171.580)	0.879
Total body	901/137656088	−85.631 (326.646)	0.797	755/116945951	−86.782 (344.681)	0.805

Model 1 adjusted for age, gender, ethnicity, smoking status, drinking status, household income, BMI, urinary creatinine. Model 2 adjusted for model 1 plus total energy intake, total protein intake, physical activity, diabetes mellitus, chronic kidney disease. Ln, natural logarithm.

**Table 4:** Odds ratios (95% confidence interval [C.I.]) of maximal grip strength fulfill the criteria of sarcopenia^*^ with one unit increase in ln-glyphosate (μg/L) in logistic regression models, with results weighted for sampling strategy.

	Unweighted sample size/population size	Odds ratio	95% C.I.		*p* value
			Lower	Upper	
Model 1	1465/210528977	1.498	0.733	3.062	0.246
Model 2	1236/180576129	1.453	0.751	2.811	0.246

Model 1 adjusted for age, gender, ethnicity, smoking status, drinking status, household income, BMI, urinary creatinine. Model 2 adjusted for model 1 plus total energy intake, total protein intake, physical activity, diabetes mellitus, chronic kidney disease. ^*^Defined as maximal hand grip strength test <30 kg in men and <20 kg in women. Ln, natural logarithm.

**Table 5:** Linear regression coefficients (standard error) of combined grip strength* per unit increase in ln glyphosate (μg/L) in different subgroups, with results weighted for sampling strategy.

	Unweighted no./population size	Adjusted *β* (SE)	*p* value	*p* for interaction
Gender				0.671
Men	588/86977749	−2.949 (1.309)	0.040	
Women	624/90724751	−1.243 (1.041)	0.251	
Age (years)				0.001
18–39	431/65089704	−0.960 (1.040)	0.370	
40–59	409/63829395	−2.118 (1.198)	0.098	
≥60	372/48783400	−3.213 (1.442)	0.042	
Ethnicity				0.393
Non-Hispanic white	585/122763359	−1.156 (1.268)	0.376	
Others	627/54939140	−3.957 (1.055)	0.002	
Annual household income (USD)				0.470
≤4,500	541/64339871	−3.569 (1.451)	0.027	
>$4,500	671/113362629	−1.185 (1.230)	0.351	
Body mass index (kg/m^2^)				0.004
<25	365/54007765	1.538 (1.228)	0.230	
25–30	382/54834733	−3.203 (0.724)	<0.001	
≥30	465/68860002	−3.220 (1.626)	0.066	

Adjusted for model 2.

## 4 Discussion

Our research utilized a sample that represents the wider adult population of the United States and has brought to light a substantial negative correlation between levels of glyphosate in urine and grip strength. However, our discoveries do not find an association between glyphosate levels, lean muscle mass, and the likelihood of reaching maximum grip strength meeting sarcopenia criteria. Our finding offers preliminary clues of a possible connection between glyphosate and muscle strength among adults in the general population. The significance of this research lies in the extensive and thorough dataset obtained from the NHANES, as well as the incorporation of a diverse cross-section of American adults.

Within this study, a remarkable 80.2% of the participants were identified as having measurable levels of glyphosate, with a mean concentration of 0.40 μg/L (4). France had a higher prevalence, with traces of glyphosate in the urine of 99.8% of the population, and an average concentration of 1.19 μg/L (32). Portugal surveyed adults from the general public and found an average glyphosate concentration of 0.1 μg/L, and a detection rate of 73% (33). A comprehensive review of 19 studies concluded that the usual glyphosate levels in urine samples from the general population were generally below 4 μg/L (34). Considering that glyphosate has a reported half-life of elimination of 5.5–10 h (35), the high detection rate implies that there are unknown and unavoidable sources of exposure to glyphosate during daily activities, which have not been evaluated by any global regulatory agency. Glyphosate residues have been detected in various food samples, including fruits, nuts, cereals, and vegetables (36). Recent research suggests that individuals with heightened glyphosate exposure may have consistently consumed foods contaminated with herbicides (37). The current study disclosed a significant elevation in glyphosate levels when adjusted for urinary creatinine among women, older individuals, non-Hispanic whites, and non-smokers. It is plausible that these subgroups had a greater consumption of such contaminated foods.

Numerous experiments have been undertaken to explore the impacts of glyphosate and GBH on skeletal muscle. A decrease in muscle strength during glyphosate exposure can be attributed to an impairment in energy metabolism. For instance, a study found that exposure to 1 or 10 mg/L concentration of glyphosate led to a notable reduction in the energy reserves in the muscles of *Odontesthes bonariensis* (10). Another study indicated that exposure to 18 μg/L concentration of glyphosate resulted in elevated energy expenditure, and a reduction in the levels of glycogen and triglycerides in the muscle of bullfrog tadpoles (9). Researchers have also investigated the effects of glyphosate treatment on muscle morphology and morphometry. In one study, the administration of water containing 0.5% glyphosate to pregnant C57BL/6 mice during both pregnancy and lactation periods did not reveal statistically significant differences in the morphology of muscle fibers and connective tissue (15). However, in another study focusing on the male offspring of these mice, a decrease in neuromuscular junctions, along with an increase in fibrosis, was observed in the soleus muscle (12). Additionally, one study examined the effect of chronic oral glyphosate (10 μg/kg for 30 days) on muscle strength in rats. The muscle contraction power decreased to 41% of the control values (13). In summary, glyphosate exposure may reduce energy reserves, alter muscle morphology and function, and reduce muscle strength. However, these studies have yielded inconsistent results, and further research is necessary.

It has been known that a decrease in neuromuscular function and a loss of motor neurons will reduce muscle fiber size and performance (38). Researchers have also explored the impact of GBH on neuromuscular junctions. In one study, exposure to 0.5 mg/L concentration of GBH inhibited muscle acetylcholinesterase enzyme activity and increased oxidative stress levels in *Cyprinus carpio* (11). However, another study involving male zebrafish showed that the activity of acetylcholinesterase remained unchanged in the muscles exposed to 5 or 10 mg/L of glyphosate during the first 96 h. Instead, there was an observed increase in the expression of lipid peroxidation levels (14). Numerous animal reports have also explored the impacts of glyphosate or GBH on the nervous system. In addition to their effects during early developmental stages (39), exposure in adulthood can induce significant alterations in both the structure and function of the nervous system (40, 41).

Most studies examining the effects of glyphosate on human health have primarily focused on the consequences of intoxication (42–44). Current epidemiological research suggests that exposure below tolerable levels is unlikely to result in adverse health effects. Nevertheless, the impact on skeletal muscles falls outside the scope of past investigations (45, 46). While there have been no previous epidemiological reports investigating the effects of glyphosate/GBH exposure on skeletal muscle, emerging research has indicated that glyphosate exposure may result in neurotoxic effects. In one occupational study, a positive correlation was found between the use of glyphosate and olfactory impairment (47), whereas another study reported a positive link between glyphosate exposure and macular degeneration (48). However, a prospective study involving Chinese farmers did not uncover any significant association between glyphosate exposure and an elevated risk of health issues, including abnormalities in nerve conduction (49, 50). Utilizing NHANES data, the current research identified an inverse relationship between glyphosate levels and hand grip strength. Additionally, the maximal grip strength threshold used in the study (maximal hand grip strength test <30 kg in men and < 20 kg in women) may not have been sensitive enough to detect differences in grip strength between individuals with varying glyphosate levels. If a causal link between glyphosate levels and hand grip strength is established, it could potentially lead to adverse effects on skeletal muscle among American adults exposed to glyphosate and GBH. Evidence suggests that glyphosate and GBH may have detrimental effects on skeletal muscle through various mechanisms, including neurotoxicity, interference with acetylcholinesterase enzyme activity, depletion of energy reserves, alteration of muscle morphology and function. Another possible explanation is that glyphosate exposure may indirectly affect hand grip strength by disrupting the gut microbiome. Glyphosate has been demonstrated to disrupt the composition of the gut microbiome in both animals and humans (51, 52), and some studies have suggested that changes in the gut microbiome can affect muscle function (53). Disruption of the gut microbiome could potentially lead to inflammation and oxidative stress in skeletal muscle, which could impair muscle function and reduce grip strength.

Our study revealed a detrimental interaction between age and glyphosate—specifically, as age increased, the negative impact of glyphosate on combined grip strength intensified. Existing literature suggests that age could play a role in influencing the sensitivity and vulnerability of the nervous system to glyphosate. Older individuals may exhibit lower levels of neurogenesis, neuroplasticity, and neurorepair, coupled with higher levels of oxidative stress and inflammation. These age-related factors may exacerbate the neurotoxic effects of glyphosate (54, 55). A similar correlation was observed in our study within skeletal muscle. Additionally, we noted that this association was more stronger in individuals with higher BMI. Several potential explanations exist for this finding. For example, research has shown that glyphosate can disrupt the gut microbiome, leading to increased inflammation and oxidative stress in the body (51, 56). This may have a greater impact on individuals with higher BMI, who are already at a higher risk of developing inflammation and oxidative stress-related diseases. As of our current understanding, this study represents the first instance in which specific demographic subgroups have been identified as potentially susceptible to the deleterious effects of glyphosate exposure on hand grip strength. Additional investigation is necessary to gain a complete understanding of the potential mechanisms underlying the observed distinctions.

It is crucial to acknowledge the study’s limitations when interpreting the results. Firstly, the study’s sample size was limited to data on glyphosate, grip strength, and DXA exams from NHANES 2013–2014, potentially constrained the feasibility of conducting a thorough analysis. Furthermore, NHANES is a valuable resource for assessing the health of the U.S. population, but it has inherent limitations as a cross-sectional study, such as the absence of detailed occupational exposure data and limited information on exposure routes and durations. Thirdly, urine glyphosate level can provide reliable estimates of actual internal human exposure that can be compared to appropriate reference values. However, the analytical methods used to measure urine glyphosate levels vary widely in terms of sensitivity, accuracy, and specificity, and that the sampling and storage conditions may affect the stability and representativeness of the samples (57). More research is needed to validate and standardize the measurement of urine glyphosate level and to elucidate the mechanisms and effects of glyphosate exposure on human health. Fourthly, the study did not consider the potential impact of other pollutants that may have been simultaneously exposed alongside glyphosate or could have affected the results. Fifthly, the age limitation of the DXA scans (18–60 years) compared to the broader age range examined in the overall study population (18 years and older) may be a potential source of ambiguity in the interpretation of the observed differences between lean mass and glyphosate. Future research should consider a more inclusive age range for relevant measurements to increase the robustness and applicability of the findings. Lastly, the study exclusively focused on adult individuals in the United States, which restricts the generalizability of the findings to other age groups and geographical regions.

## 5 Conclusion

After conducting an analysis of a representative sample of U.S. adults, our study has unveiled significant evidence pointing to an inverse connection between urinary glyphosate levels and hand grip strength. Furthermore, our findings hint at a potential negative correlation between glyphosate levels and lean body mass in both arms, although this relationship was only marginally significant. While additional research is needed to ascertain the clinical significance and causative factors behind these findings, our results underscore the importance of continuous investigation into the potential harmful effects of glyphosate on adult skeletal muscle. Such studies have the potential to inform public health policies regarding glyphosate usage, thereby contributing to the protection of human health.

## Data availability statement

The datasets analyzed during the current study are available at the NHANES website (https://www.cdc.gov/nchs/nhanes/index.htm (Accessed March 5, 2023).

## Ethics statement

The studies involving humans were approved by Ethics Committee of the En Chu Kong Hospital (ECKH_W11212). The studies were conducted in accordance with the local legislation and institutional requirements. The participants provided their written informed consent to participate in this study.

## Author contributions

Y-WF: Conceptualization, Data curation, Formal analysis, Investigation, Methodology, Software, Writing – original draft. CW: Formal analysis, Methodology, Software, Supervision, Validation, Writing – original draft. C-YL: Conceptualization, Data curation, Funding acquisition, Investigation, Methodology, Software, Visualization, Writing – review & editing.
